# Scientific experimental articles are modernist stories

**DOI:** 10.1007/s13194-024-00592-7

**Published:** 2024-07-17

**Authors:** Anatolii Kozlov, Michael T. Stuart

**Affiliations:** 1 Institut Jean Nicod (CNRS-EHESS-ENS), Paris, France; 2 Department of Science and Technology Studies, University College London, London, UK; 3 Department of Philosophy, University of Geneva, Geneva, Switzerland; 4 Department of Philosophy, University of York, York, England

**Keywords:** Scientific articles, Scientific narratives, Scientific experiments, Scientific imagination, Scientific understanding, Aesthetics in science, Modernism

## Abstract

This paper attempts to revive the epistemological discussion of scientific articles. What are their epistemic aims, and how are they achieved? We argue that scientific experimental articles are best understood as a particular kind of narrative: i.e., modernist narratives (think: Woolf, Joyce), at least in the sense that they employ many of the same techniques, including colligation and the juxtaposition of multiple perspectives. We suggest that this way of writing is necessary given the nature of modern science, but it also has specific epistemic benefits: it provides readers with an effective way to grasp the content of scientific articles which increases their understanding. On the other hand, modernist writing is vulnerable to certain kinds of epistemic abuses, which can be found instantiated in modern scientific writing as well.

## Introduction

Experimental scientific articles were once respected as important carriers of the content of science. But then they slipped out of view. One reason for this is that they were thought to be unrepresentative of real scientific practice. Yet, real scientific practice obviously includes the reading and writing of scientific papers. And this should be accounted for.

In this paper, we aim to bring philosophy of science back to experimental articles by developing an epistemology of scientific writing consistent with recent work on narrative as a conceptual lens to study science. After rejecting the widespread but implicit view that articles are merely arguments, we argue that a narrative-based view of scientific articles does better to capture the epistemic aims and features of experimental articles, and go further to claim that the techniques used to write scientific experimental articles can productively be understood via an analogy to modernist techniques in art. We close by considering how this analogy helps to explain the way that scientific articles engage imagination and lead to (mis)understanding.

## A brief history of the philosophy of experimental articles: from product, to argument, to practice

The mismatch between the actual practice of science and how it is reconstructed in scientific publications was an important motivator for Hans Reichenbach’s distinction between the contexts of discovery and justification:

The way, for instance, in which a mathematician publishes a new demonstration, or a physicist his logical reasoning in the foundation of a new theory, would almost correspond to our concept of rational reconstruction; and the well-known difference between the thinker’s way of finding this theorem and his way of presenting it before a public may illustrate the difference in question. (Reichenbach, 1961 p.6)

Reichenbach is correct that scientific research papers “recast the events, replacing the actual steps that were undertaken with operations that can be demonstrated as valid”, for example, by changing the temporal order of the research activities, “rationalizing” motivations for doing certain experiments, by reorganising the data sets, and so on (Schickore, 2008). As Schickore notes, this observation became a starting point for almost all scholars of science at the time. For example, scientist and philosopher Medawar (1963), taking scientific reports as written in the inductivist mode (an influence he attributed to John Stuart Mill), famously complained that in the contemporary form, the scientific paper is a “fraud”. As a result, he advocated for more faithful representations in scientific publications of the trajectory of research. Further, lab anthropologists such as Gilbert (1976), Knorr-Cetina (1981), Latour and Woolgar (1986), and Latour (1987) reinforced Reichenbach’s distinction between the contexts of discovery and justification. According to Knorr-Cetina, scientific articles are written “with a view toward potential criticism or acceptance (as well as with respect to potential allies and enemies!)” (1981, 7). In other words, scientists write articles with the aim of persuading their peers and winning sympathy for their interpretations. A similar framework was taken up by historians of science, many of whom analysed the literary structure of scientific arguments (e.g., Dear, 2015). Shapin (1984) explicitly analysed Robert Boyle’s literary strategy for communicating experimental “matters of fact”, calling it “virtual witnessing” catered for a “genteel” audience. Bazerman (1988), Gross (1990), Atkinson (1996), and others collected rich material on the literary underpinnings of scientific argument, at the same time added fuel to the “Science Wars” by stating that “the claims of science are solely the products of persuasion” (Gross, 1990, p. 3).

Scientific articles were a forgotten casualty of the Wars, until 1998 when the discussion was re-launched with a paper in *Philosophy of Science* by Lipton (1998), which characterized scientific articles as centrally employing Inference to the Best Explanation (IBE), while Franklin and Howson (1998) championed a Bayesian approach. Using a famous case from geology (Morgan, 1968), Suppe argued that neither hypothetico-deductivism (HD) nor Bayesianism, nor IBE, could satisfactorily account for the structure of scientific experimental articles (Suppe, 1998a, b), pointing out that scientific articles are so severely constrained by the demands of scientific journals (e.g., length requirements) that each and every element of the article – paragraph, diagram, table – must contribute to the justificatory argumentative structures of the claims made in the article. While Bayesianism or IBE might account for some *minor* parts of scientific experimental reports, the rest of the content remains unaccounted for. According to Suppe, many of the scientific knowledge claims in the articles are not ampliative; beyond that, there seem to be two options: either the justificatory schemes of scientific experimental articles are different and more diverse than justificatory models proposed by philosophers, or scientific articles do not aspire for justification in a strong sense. Indeed, as Suppe (1997) observes, experimental reports “present the reduced data or results of the experiment”, make “an interpretation of the reduced data (results) which yields the specific experimental claims”, but for the most part are “descriptive, not argumentative”. Hardcastle’s (1999) follow-up to Suppe’s work offers counterexamples to Suppe’s rejection of HD, showing that evidential claims in scientific works of Kluck et al. (1997) and Chinnaiyan et al. (1997), can, in principle, be mapped onto HD; at the same time she insists that these two mappings differ drastically between themselves, and the presence of HD traits in no way proves that this argumentative strategy will be instantiated broadly. Hardcastle advocates that “a gentle and ecumenical pluralism more accurately reflects scientific reality”, concluding that “the purposes of the articles drive their argumentative structure”.

Suppe’s and Hardcastle’s analyses show that the argument schemes in scientific reports are much more evasive, implicit, and goal-dependent than one might expect, something that accords with Reichenbach’s own initial observations:

Even in the written form scientific expositions do not always correspond to the exigencies of logic or suppress the traces of subjective motivation from which they started. (Reichenbach, 1961, p. 7)

Furthermore, it is possible that many experimental reports are not even concerned with mounting a distinct theoretical argument: exploratory research is a case in point. And even if scientists read research reports partially as arguments for some conclusions, they may not necessarily agree on the exact conclusion to be taken away from them (see Gilbert, 1976). It is also important to note that scientific papers can (and do) tell readers much more than which conclusions are justified by the new data. They tell what is being assumed, done, and observed by their authors, not merely to serve as premises for a conclusion, but also as means of telling the reader where the scientists *may* be going wrong, and what was *not* done, to allow the reader to imagine *what else* might have been done instead, to offer up for analysis certain results that might be built upon, or which might be in error, to suggest other avenues for research, and so on. These are genuinely epistemic functions of scientific reports that practising scientists recognize.^1^

One might parry that these functions *can* be captured by a view of papers merely as arguments. All the additional information just pointed to could be characterized as forming part of counterfactual reasoning that might be done by the reader, as it were, on their own time. But such counterfactual reasoning happens *outside*, not *inside* the scientific paper. The paper merely ‘affords’ counterfactual reasoning and cannot be identified with that reasoning. This renders scientific articles *vehicles* for scientific reasoning rather than arguments themselves.

Since the epistemic function of experimental papers cannot be exhausted by characterizing them as arguments, we suggest looking closer at their epistemic means and ends.

## Experimental reports are data-driven and open-ended

What do experimental reports *do*? The obvious answer is that experimental reports report experiments. Well, then: what do experiments do? The epistemology of experiment was a neglected topic for a long time in philosophy of science, but once it took off, a central refrain has been that experiments have a degree of autonomy from theory: “Experimentation has a life of its own”, as Ian Hacking says (1983, 150). The exact degree of autonomy is a matter of debate, but whatever autonomy experiments have, it will at least partially be due to its having aims other than testing theoretical hypotheses, including validating experimental systems or exploring noteworthy phenomena. Indeed, Rheinberger (1997) has argued that most experiments in biology do not pursue explicit theory testing.^2^

Another centre of gravity in the epistemology of scientific experiments has been *materiality* (Rheinberger, 1997; Harré, 2003; Radder, 2009). Experimental systems can usually be divided into *objects* of experimentation, treatment, and intervention; and *targets* of experimentation, e.g., the phenomena scientists intend to learn about via their experiments (Currie, 2018; Parke, 2014; Winsberg, 2009). In practice, the distinction between the two might be not so neat, as when the behaviour of a particular experimental system is investigated, making the target and the object the same thing. But in any case, materiality is thought to be epistemologically important when it comes to how insights about experimental objects can generalize to targets outside the lab. Other concerns related to materiality include questions about the stability of the experimental system, reproducibility, and internal and external validity (Radder, 1996, 2009). The materiality of measurement instruments is also important. Scientific articles aim to present experimental data that enables inferences to be drawn about the reproducibility of findings, as well as information about the internal and external validity of methods used. But these are best evaluated when the precise conditions of experimentation are known, so we end up with a formula for the report that involves descriptions of *what* was done, and *how* (including material details about the instruments and prepared materials), and *what* the outcome was.

In sum, partially autonomous data and the material of their production are the foundation of experimental reports. Since scientists cannot know in advance the outcome of their experiments, and sometimes they might not know in advance the very experiments they are going to do (the possibilities unfold along the way), it only makes sense that the reports are written after the experimentation takes place. Thus, experimental reports, as the experimental investigations themselves, are outcome-driven, as they bring together and try to make sense of sets of experimental observations. This autonomy of experimental outcomes from initial epistemic motivations (as well as theoretical frameworks) also puts a strain on the evidential use of the experimental outcomes: scientists might discover that their research is not about what they thought it was going to be about.

This continues to be a source of confusion for everyone learning to do science, as individual experimental reports look as if scientists knew from the beginning what they were up to (Schickore, 2008; Meunier, 2022; Diaz Gonçalves, 2023). However, this is a necessary byproduct of experimental research: the concluding interpretation of the results can occur only after they were obtained; the latter helps to identify the exact epistemic gaps these results might be filling, and suggest what background literature would be helpful for interpreting them. A little vexation that comes at this point is that scientists, in their introduction sections, sometimes ascribe to themselves to a semi-fictional motivation to answer *that particular* question. From a certain point of view, such misrepresentation of the research process might look like epistemic wickedness, however, one alternative interpretation is that it facilitates readers’ understanding of the results and thereby is tolerable insofar as the core content of the paper remains factive.

The question now is: how can scientists present their work such that this data-drivenness and open-endedness are captured and represented along with the data and analysis, such that all of this can be made epistemically useful to others in a short space? We think that in response, scientists have adopted something like the set of *modernist* narrative conventions, which, as we will see, are open to interpretation, factive, and able to present different perspectives at the same time.

## Stories and science

A body of recent philosophical work heralds a revived focus on the uses of narrative in science.^3^ Narratives are discovered at various sites of scientific practice, especially in idiographic sciences such as history and natural history (Currie & Sterelny, 2017; Terrall, 2017), but also in mathematical simulations and modelling (Rosales, 2017; Wise, 2017), sociology (Morgan, 2017), clinical case reporting (Hurwitz, 2017) and thought experiments (Murphy, 2020; Nersessian, 1992, 2017; Swirski, 2006; Stuart, 2021). Philosophers argue that narratives can do many things, including: explain (Roth, 1989); demonstrate the pursuitworthiness of a model (Hartmann, 1999); capture complex causal connections (Morgan, 2017); identify gaps in knowledge (Currie & Sterelny, 2017); provide causal mechanistic explanations (Swaim, 2019); order knowledge, provide coherence, and exemplify scientifically important features (Morgan & Wise, 2017; Kranke, 2022; Haines, 2022); as well as operate as a form of counterfactual explanation (Beatty, 2017). The intensive work on narratives recently culminated in a volume on narratives in science, edited by Morgan et al. (2022), which characterizes narrative as a general-purpose “technology of sense-making” (p. 4).

But what about scientific papers *themselves*? Out of the twenty-two entries collected in Morgan et al. (2022), only two, Meunier’s (2022) and Jajdelska’s (2022), explore the narrative aspect of contemporary scientific papers. We understand this omission as one consequence of the “turn to practice”, in which this new literature on narrative places itself. As mentioned at the start of this paper, scientific papers (as well as textbooks) were the main source of information upon which philosophers of science drew, as they were thought to contain the “output” of science. These were the explananda for positivistically-inclined philosophers, whose main aim was to ground this output in terms of pure sense-experience. The turn to practice de-centred scientific papers as the locus of philosophical attention by urging philosophers to peek their heads behind the curtain, and see how the magic was really done. For example, while Rouse (1990, 2018), a leading figure in the turn to practice, characterizes science as a “narrative in construction” through which science acquires its intelligibility and significance; he excludes scientific research papers from being narratives themselves, in favour of the view that scientific papers are merely arguments. This change in orientation was central to the turn to practice, and still underlies much work in philosophy and sociology of science (for an overview, see Schickore, 2008). Thus, apart from a few recent works (Hughes, 2006; Meunier, 2022; Jajdelska, 2022; Pomata, 2014; Hurwitz, 2017) and scattered remarks here and there (Feyerabend, 1991, 493; Feyerabend, 1995, p. 163), philosophical reflection about the narrative nature of scientific articles is limited to the analysis of scientific writing *in general*, or in the context of some particular historical cases (Rheinberger, 2020; Terrall, 2017; Wise, 2020).

At the same time, the view in philosophy of art seems to be that scientific articles aren’t narratively or literary interesting. For example, Derek Matravers agrees with Kendall Walton’s view that ‘many or most books on science, technology and engineering,’ along with recipe books and instruction manuals, do not require imagination because “they are not narratives” (Matravers, 2014); while Arthur Danto holds that the literary dimension of scientific writing (e.g., in physics) “must seem deeply secondary” (Danto, 1986, p. 136).

And yet, story-talk is a pervasive part of scientific *practice*: in presenting research results, at a conference or in a manuscript, scientists often explicitly aim to “tell a story”. Theoretical chemist and Nobel Prize winner Roald Hoffman is clear:

Having read thousands of chemical papers and listened to hundreds of colleagues’ lectures, I chafe against being ruled out of bounds. In the papers I read and write, I feel stories unfold before me. I react to them emotionally. I sense narrative devices in these articles and lectures, employed both spontaneously and purposefully. (Hoffmann, 2017)

Many writing guides for science students explicitly advocate for a form of storytelling in their papers, as “good” stories have a higher chance of being well-received by peers and publishers (see, e.g., Tomaska, 2007; Gemayel, 2016; Mack, 2018; Villar, 2020). A dispute that happened on the pages of *Nature* in 2013 brought the question into explicit focus. In an attempt to stimulate more engaging writing, Krzywinski and Cairo argued for an analogy between scientific research and stories (Krzywinski & Cairo, 2013). They proposed that in presenting data, scientists could “use the idea of a story arc”:

Maintain focus of your presentation by leaving out detail that does not advance the plot. Distinguish necessary detail from minutiae; do not give in to the desire to show all your hard-won data. Provide sufficient support for your story, but stick to the plot.^4^

In the rest of this paper, we explore the narrativity of contemporary scientific articles. By narratives we mean perspicuously ordered representations of at least two events that are unified in a forward-looking manner, concerning some scientific objects of study, that transport the reader away from the here and now by causing readers to imagine engaging with an author who constrains themselves to present things mostly as they believe them to be. We will also assume that narratives have an emotional arc^5^. In our inquiry, we will be specifically focusing on the case of experimental articles, i.e., ones that report experimental findings and experimental data produced with the help of multiple experimental methods and instruments.

## Experimental reports narrativize perspectives

It is common knowledge that contemporary experimental reports are frequently presented in Introduction, Methods, Results and Discussion sections (IMRD). Meunier (2022) looks at IMRD sections as epistemic *scenes* through which the reader engages with the report. He suggests that experimental reports contain two distinct narratives: a *narrative of nature*, that recounts how nature ‘works’; and a *research narrative*, which tells what scientists did in order to obtain those results. According to him, the research narrative is necessary for the reader to grasp the experimental procedure and evaluate the possible hypotheses at stake before the conclusions are accepted as part of the narrative of nature. We agree with Meunier’s emphasis on the role of narrativity in allowing readers to grasp the reported experimental procedures. We want to take it further. For one, what sort of narratives are the narratives of nature and the research narrative? To answer, we follow Suppe’s (1998a, b) advice to look at the *microstructure* of papers.

To choose an example at random, the microstructure of the Results section in Liang et al. (2016, 2017, 2019)^6^ reveals an interesting correspondence between the things told and the things shown. What we notice is that the results of each section are given through a recurrent linguistic pattern of researchers encountering nature via a particular technique. Consider the following excerpt:

[*] If these Ca^2+^ rhythms are critical output features of M and E cells, their properties may also reflect differences in photoperiodic entrainment. We entrained flies under either long-day (16 hours light, 8 hours dark) or short-day (8 hours light, 16 hours dark) conditions. In these flies, the phase difference between the morning and evening behavioral activity peaks tracked dawn and dusk (fig. S6). Likewise, the phases of pacemaker Ca^2+^ rhythms also tracked dawn and dusk (Fig. 3, A, B, E, and F, and fig. S7). Regardless of the photoperiodic schedule, the s-LNv (M cells) always peaked around dawn, whereas the LNd (E cells) always peaked before dusk (Fig. 3, B to D and F to H). Thus, Ca^2+^ activity patterns within the pacemaker network correspond to the circadian temporal landmarks of dawn and dusk. (Liang et al., 2016).

Here we find *motivation* (locutions “to study how…”, “to test whether…”, etc.), *action* (“we measured“, ”we removed”), *observation* (“the rhythm peaked”), and *conclusions* (“taken together, these results show”).

The first thing to note is that the actions and observations often come as shifts between the perspective of the *researchers* (“WE measured”) and the perspective of *nature*, or, objects of study (“CELLS peaked”). These shifts are not narratively and epistemically idle, since they capture the gap between the researchers’ actions and nature’s responses. For the reader, it wouldn’t matter that the intentions might be the authors’ retroactive “rationalisations”, since the transition from not knowing to knowing nature’s response to the factual experimental situation is inevitably suspenseful; it also may elicit emotions similar to those elicited by experiments themselves (Kozlov, 2023a, b; Morgan, 2005; Currie, 2018). This will be important to remember for discussion (below) on the epistemology of *reading* experimental reports.

The second observation concerning the above excerpt is that despite some variations, these narrative patterns pave the Results section of all three papers, and in most cases, they also refer to a particular piece of data that the reader can inspect by themselves on the figure panels, where the number of figures counts in the dozens. Thus, the three papers just mentioned (Liang et al., 2016, 2017, 2019), excluding supplement materials, contain about forty to sixty lettered figures (several letters per panel) often with several sub-figures^7^. Each of these is a piece of different data or a diagram in various formats. Each stands for a glimpse given by researchers through a particular experimental lens at the particular object of study. Recently, the idea of epistemic perspectives gained particular traction in philosophy of science (Giere, 2006; Van Fraassen, 2008; Massimi, 2022), and we think aspects of this notion are useful in the present context. Thus, Giere (2006) developed a concept of epistemic perspective rooted in scientific instruments:

In the most general sense, scientific instruments are perspectival in that they respond to only a limited range of aspects of their environment…The relationships between inputs and outputs always remain to some extent a many-one relationship. The nature of this relationship is part of the perspective of any particular instrument. (p. 41–42)

In our case, each mini-narrative of each experimental report is based on an individual instrument-based perspective. Further, what is observed from that particular instrumental perspective, in line with van Fraassen’s (2008) notion of *indexical judgement*, is subject to aberrations, analogical to occlusion or marginal distortion in perspectival visual representations. This raises concerns about the limitations of individual observations, which must constantly be kept in mind. In line with Massimi’s (2022) epistemic perspectives as *vanishing points*, each observation via a particular situated perspective opens up a line of epistemic possibilities and research implications, and for researchers with different epistemic backgrounds, these implications will be different.

This brings us to a key point. Experimental reports gather together sets of instrument-based observations that are somehow grouped under a common theme, claim, or statement. To make sense of them, researchers need to integrate together different perspectives as well as integrate them with existing background knowledge (Gonçalves, 2023). One way to understand this process is through what Morgan calls narrativizing (Morgan et al., 2022):

Narrativizing is a way for scientists to organize their bits of scientific knowledge to create sense out of their relations. Narrativizing serves to join things up, glue them together, express them in conjunction, triangulate, splice/integrate them together (and so forth). Yet, the need to clarify relations between things means that narrativizing sometimes means scientists have to sort things out so that their interrelations can be seen more clearly. (p. 12)

According to Morgan, two ways of narrativizing are *colligation* and *juxtaposition*. Colligation “brings together, and assembles, a set of similar elements framed under some overall guiding conception, or categorization schema”; the resulting narrative is an instrument of ordering and coherence-making and it emphasizes relations between the elements that feature in the narrative. In contrast, juxtaposition leads to disjunction-based narratives, in which “many elements initially presented … don’t appear to fit together”. Disjunction-based narratives are *question-raising* tools, which call attention to incongruencies and conceptual puzzles, forcing one to rethink the relations between the juxtaposed elements. Morgan uses the analogy of visual art to explain how this works: the juxtaposition of elements, just like the juxtaposition of perspectives in paintings, highlights the epistemic gap between the different elements to be filled in by the readers or spectators (Morgan, 2017).

Experimental reports use both techniques, colligating a great deal of complimentary information together while juxtaposing the perspectives from which that material was obtained. Scientists often present their favourite interpretation of this mix of similarity and difference in the discussion sections of their papers, in a hypothetical way that leaves it open for the reader to develop other interpretations. By grouping observations and juxtaposing perspectives, they present a web of possibilities for what might be done or tested further, depending on who is reading it. They can be seen as a “canvas” of multiple facets through which researchers can grasp not just the experimental procedure itself, as Meunier (2022) suggests, but also the epistemic landscape underlying the reported observations.

By highlighting the roles of colligation and juxtaposition, the narrative view of scientific articles allows us to recover insights of earlier philosophers of science writing on the topic. For example, Suppe, looking at experimental articles, insists (contra Kitcher, 1991) that even if some of their parts are rhetorical, they are also epistemic insofar as they disclose the processes of “cutting up the data”, “isolating causes”, and “eliminating alternatives” to be found in on-going experimental research (Galison, 1987, p. 258). We see a similar thought expressed by Mary Morgan concerning the epistemic role of narratives in science (2017):

[Narratives] find ready space in making sense out of mathematical simulations in the natural sciences and economics, in giving accounts of chemical reactions, and in counterfactual approaches in political science. These are sites in which scientists get to know things via narrative, not because the narrative provides an illustrative example for theories or models or something else, nor because it is ‘merely’ rhetoric (*though rhetoric is never* ‘*mere*’), but because narrative is how the relationships amongst their materials become known to them. (p. 87, italics added)

One way that philosophers of science characterize the epistemic power of narrative is in terms of facilitating a grasp of a subject. Narrative does this by imposing “orderliness” on its contents. There are many examples discussed in the literature. Let us consider a few.

Currie and Sterelny (2017) demonstrate how in historical sciences the construction of narrative explanations allows overcoming local causal underdetermination via a gradual increase of stringency, coherence, and scope of explananda and explanans. They also note how the combination of narratives and simple formal models helps to explain “highly complex historical sequences”.

Morgan (2017), on the other hand, illustrates how explanatory narratives in social sciences emerge from the process of *configuring* (see also Cristalli, 2019). Arranging and putting together various research resources – conceptual, theoretical, evidential, empirical – helps to create a coherent epistemic narrative that allows the reader to make sense of the phenomena involved.

These and other examples jointly gesture toward the fruitfulness of a narrative-based account of the epistemology of scientific reports, which we develop in the next sections.^8^

## A narrative epistemology of scientific articles

If experimental reports are narratives, how do they work? Most straightforwardly, we can rely on Gricean insights about the authorial intentions which cause certain interpretations in the minds of readers, via certain social conventions. When speaking of writing, many of those conventions fall under what we might call the “genre” of the work. In what follows, we assume that the “epistemic genres” of science (Pomata, 2014) can be illuminated by reference to work on aesthetic literary genres. As Shen-Yi Liao notes, citing Todorov (1990), genre has two functions: shaping the readers expectations, and providing models for authors to emulate (2016, 469). Genres are descriptive classifications of narratives, but they are also normative, in the sense that they tell the audience what to expect and how to react, including what to take as true in the fiction (Liao, 2016, 470). Specifically, we will argue that in their epistemic function, experimental reports in the hard sciences can be fruitfully characterized as *modernist*.

In the domain of visual art, Cubism is a paradigm instance of modernism. It famously questioned the artistic convention of emulating visual experience from a single point of view. Instead of imitating visual experience from one vantage point, cubist paintings collocate the appearance of an object from a multitude of perceptual points of view simultaneously, which was made possible by the fragmentation of the depicted object into various geometric figures. Art critic Jacques Revière, defending the value of Cubism, frames this in an epistemological way:

Certainly reality shows us these objects mutilated in this [perspectival] way. But in reality we can change position: a step to the right and a step to the left complete our vision. *The knowledge we have of an object is*…*a complex sum of perceptions*. (in Fry, 1978 p. 77, italics added)

By combining perspectives in a stark, unnatural way, Cubists were able to present reality in a way that was, in a sense, *more* accurate. And this was an explicit choice: “When Braque and Picasso found their work approaching the non-representational or non-figurative or non-objective (all these terms are used), both artists ‘recoiled.’ They chose, like Cézanne and Matisse and the great majority of post-impressionist and modernist painters, not to lose sight of the object. For this reason among others it is often said that the aim of Cubism was essentially to represent reality more accurately and completely” (Vargish & Mook, 1999, 129).

Cubist treatments of the visual medium found a counterpart in the literary work of modernist authors like Virginia Woolf, James Joyce, Gertrude Stein, and others, who gained prominence by assembling fragmented and apparently disconnected episodes into single narratives. Whereas cubist painters overcame the limitations of a single point of view, these writers overcame the canonical linear narrativity of episodes and stories (Isaak, 1981; Lacourarie, 2002; Doss, 2003; Falcetta, 2007; Weiss, 2012). As Cubism played with the juxtaposition of frames, perspectives, formats, and media, the new narrative style played with temporal and stylistic non-continuity and non-linearity, for example, through a *montage* of perspectives and episodes taken from narratively non-adjacent spaces and storylines. The rupture of the storyline is characteristically marked by sudden shifts in the narrative point of view (“focalisation” in narratology). In some cases, the transition between the perspectives was linked via a particular entity seen by different characters. For example, in *Mrs. Dalloway*, in the episode when Clarissa Dolloway arrives at the flower shop of Miss Pym, we see an explicit assembly of perspectives and characters, when there is a sudden “violent explosion” outside of the shop, which startles both Clarissa and Miss Pym; from this we instantly follow how the cause of this noise (a motor car) is perceived by: some “passers-by”, by a certain Edgar J. Watkiss (we won’t encounter him ever again), by Septimus Warren Smith and his wife Rezia, and finally, by the future “curious antiquaries, sifting the ruins of time”; right after that we observe even more spatially distanced characters unified by their sight of the aeroplane disappearing and reappearing in the sky: it is being noticed by Mrs Coates and Mrs Bletchey in front of Buckingham Palace, again by Rezia, now at Regent’s Park, by Mr Bentley “vigorously turning his strip of turf in Greenwich”, by a seedy-looking non-descript man at St. Paul’s, and finally by Clarissa herself, now entering her home thinking “What are they looking at?” (Woolf, 1925).

Recent trends in aesthetics and philosophy of literature attempt to see the epistemic import of literary texts, fictional and non-fictional, beyond trivial considerations of their truth claims (Mikkonen, 2015b). In this vein, modernist narratives have gained a reputation as particularly “epistemological”: they tend to foreground questions related to perspective, cognition, and the reliability of knowledge, and they often explore and expose how perception, action, and thinking are “inextricably linked” (Miguel-Alfonso & Mikkonen, 2020; Verheyen, 2018). The stark juxtaposition of narrative perspectives, plot scenes, and discursive styles, at each of these levels, works precisely towards that: it brings to the readers’ attention the issues of the narrative’s constructed and interpretative nature while attempting to present reality (or a model of it) in a more complete, though more complex, way. The juxtaposed elements foreground the epistemic gaps between them without explicitly spelling out the links. For one, this can compress the discourse by omitting obvious or repetitive information. But it can also be used when the explicit link is not known or there are multiple possibilities for such a link. Either way, in order to make sense of the story or to enhance its aesthetic effect, the encountered juxtaposition invites readers to fill in the gaps *themselves* using their own imagination, knowledge, and experience. For example, this is how screenwriter David Mamet (2002) describes the *montage* in modernist cinema:

[Montage] meant the juxtaposition of two disparate and uninflected images in order to create in the mind of the viewer a third idea, which would advance the plot. (A man who’s walking down the street turns his head and reaches tentatively to his pocket; shot of a store window with a sign that says SALE; the viewer thinks “Oh, that man would like to buy something.”) The first idea juxtaposed with the second makes the viewer – us – create the third idea.^9^

Naturally, the failure to fill the exposed gaps can lead to confusion and failure to interpret the narrative altogether. Mikkonen (2015a) maintains that forms of confusion furnish the cognitive value of literature: “Confusion makes us test and revise our conceptual resources. If our conceptual resources prove insufficient in explaining a given phenomena, the resources might be reassessed” (pp. 123–136). Interestingly, Verheyen (2018) argues that, while fiction, in general, invites interpretation and simultaneously resists a single interpretation, modernist literature opens things up still further. He suggests that the readers’ role in this context is not to decipher the referents concealed behind the fictive story, but to produce *new* meanings, and discover new possible referents using their productive imagination.

Something altogether similar can be said about experimental reports, though here the elusiveness of the referent stems not from the authors’ literary intentions, but from the nature of the inquiry and epistemic (instrumental and conceptual) complexity involved in representing the subject of study. As mentioned before, experimental reports weave together the perspectives of researchers and objects of interest, where the essential point of contact between the two perspectives is the instance of scientific observation. Furthermore, reading the experimental narrative we follow the observations that refer to real-life events in the research process; however, what eventually presents itself as a streamlined narrative in the article, in the preceding research process refers mostly to spatiotemporally distinct events: repeats and variations of different experiments, each of which often involves preparations of new samples, instruments, and measurements. Thus, in reading such research narratives we must remain alert to the contingencies and particularities of the material epistemology of experimental practice and the different theory- and instrument-based perspectives involved. With the images, diagrams, and plot at hand, we exercise our own judgment and decide whether we agree with what the research narrative suggests. Thus, in reading these narratives, and following through the figures, we ultimately *cannot* be passive. In order to make sense of the reports, we are continuously invited to re-trace the missing narrative links and fill in the gaps between the juxtaposed perspectives using our imagination, given whatever background knowledge and research experience we have. With all this, the reports are rewarding as narratives for two reasons: first, the “dramatic” structure of epistemic set-ups (experiments) and payoffs (observations), where the latter unfold like events in a drama^10^, and second, due to the exercise of imagination required. For the prepared reader, the reported experimental observations may easily turn out to be surprising, confusing, awe-inspiring, curious, worrisome, in other words, emotionally stimulating, which by itself can navigate the readers’ attention to the aspects of reported observations in an epistemically productive way (Kozlov, 2023b).

## Epistemology of experimental reports: understanding through imagination

On the one hand, scientific experimental reports can be seen as sets of experimental claims, although not necessarily as forming a specific overarching theoretical argument. On the other, these reports can be seen as narratives that communicate what is being assumed, done, and observed by their authors; and convey where the scientists *may* be going wrong, and what was *not* done; in the latter case, they come forth as vehicles for exploratory reasoning, allowing the reader to “make sense” of what is being reported. This suggests a dual view of the epistemology of experimental reports: one concerned with the epistemic reliability of individual statements, and the other with the overall understanding that the report affords. Such a dual view seems consistent with the ambivalent attitudes experimental scientists have towards the results of their experimentation. As Kozlov (2023b) observed, experimentalists, attending to their results, take up an interestingly cautious attitude: they believe individual claims, but simultaneously look for confoundments of their observations and reflectively explore the results’ holistic implications. Given that scientists can be expected to take a similar attitude towards published experimental narratives written by others, we think our analogy with modernism lands well here, at least in the sense that it captures important epistemic and aesthetic features of reading and writing experimental articles.

It is not new to claim that narrative, fiction, or genre conventions can be understanding-conducive (Mikkonen, 2015b, 2021; Elgin, 1993). The idea goes back at least to Max Weber (Do Valle, 2022; Turner, 2017), but possibly further to Aristotle (Velleman, 2003). For Velleman, “how storytelling conveys understanding is inseparable from the question what makes for a good story” (2003, 1). In what follows, we will suggest that the increase in understanding is mediated through acts of imagination. Angela Breitenbach (2020) writes, “artworks as well as [scientific] theories strike us as beautiful when they force a range of imaginative activities on us” (79). This necessary involvement of imagination is characteristic of modern art. In watching a modern dance performance, Breitenbach reports that she is not there to decipher “a fixed code”, but rather to draw out ideas that were suggested, but “never made fully explicit, by the artwork itself”. The beauty lies in appreciating “ideas of great significance whose content went beyond any particular representation of what was shown by the dance itself” (73). And the very same thing happens in science: “a theory…may force us to imaginatively draw out the specific aspects of those situations, and to examine them in relation to other theoretical principles we hold true and take to be important. This imaginative activity is a further part of our ability to achieve and deepen understanding” (80).

Replacing “theory” with “scientific article”, this is exactly our point, though what we imagine might be different in the two cases: imagining with a theory might involve creating models of the theory, while imagining with a paper might involve imagining what was done. With respect to the role played by imagination, Breitenbach draws a parallel between art and science: in both cases, imagination mediates our experience of beauty and our experience of understanding. However, a natural worry arises: the mere *experience* of understanding is separable from the achievement of *genuine* understanding. Breitenbach recognizes this, and claims that the feeling of understanding is a signal that scientists are “on the right track at least in this sense: they are engaged in activities that contribute to scientific understanding” (86). We are not sure this is enough: scientists already know they are genuinely engaged in the search for understanding, and having an experience that signals this does not increase the epistemic value of their position. What we aim to provide is an account that explains how reading scientific articles genuinely increases understanding in a way that is mediated by the imagination.

We briefly consider two (mutually consistent) proposals that relate modernism to understanding via imagination. They differ principally in the kind of understanding they claim might be produced through reading.

The first kind of understanding is called “objectual understanding”, and it is typically characterized in terms of grasping the dependency relations that unite a system of information surrounding a particular phenomenon or subject matter (Baumberger, 2019; Baumberger et al., 2016; Dellsén, 2020; Kvanvig, 2003; Elgin, 2007, 2017; Wilkenfeld, 2019; Kelp, 2015; Hannon, 2020). Some philosophers, including Finnur Dellsén, Catherine Elgin and Jonathan Kvanvig, claim that this is the “core” or “paradigm” conception of understanding. The dependency relations grasped could be semantic, causal, mathematical, explanatory, or otherwise. For example, to have objectual understanding of circadian rhythms, a scientist must grasp what circadian rhythms are, what our current models of circadian rhythms are, the limits of these models (perhaps in terms of the data they can and cannot explain, and the features of circadian rhythms they do or do not explain), the methods used to produce data about circadian rhythms, and what upstream and downstream phenomena are connected to circadian rhythms.

As mentioned, philosophers who write about narrative and explanation emphasize the way that narrative modes of expression show how various items cohere (Crasnow, 2017; Rosales, 2017; Morgan, 2017; Mink, 1970). We think this is correct. To have objectual understanding, a scientist needs to see the connections between the target system, the history of work on that system, and the relevant data, methods, models and mathematics. Coherence can be produced in the mind of the scientist when all of this information is presented in a single narrative. But coherence between all this information is very difficult to achieve in modern science, given the sheer number of connections, and the varying strengths of these connections. And any false sense of coherence must be avoided, given that what seems coherent today might turn out never to have been tomorrow. By presenting information in the modernist way, scientists allow, or even *demand* that readers draw their own connections between all these elements, as well as to consider the strength of these connections by using their imaginations to probe them. This is precisely what makes the consumption of modernist art so challenging, and so rewarding. It forces the audience to fill in the blanks using their imagination, in order to produce their own image of the artistic object. And this has the side effect of making the artistic experience more personal. Scientific writing is the same: coherence is a key feature of objectual understanding, but it cannot be transmitted from writer to reader merely by text and diagram. It is an important goal, which scientists produce in themselves via their daily practice, and aim to communicate responsibly via something like modernist conventions. And those conventions are chosen given the fact they allow for a great deal of things to be quickly glanced together at once, from a number of different perspectives, in a way that demands the exercise of personal imagination.

A second proposal concerns “practical understanding”, which is typically characterized in terms of abilities that are relevant to (or constitutive of) understanding. This is perhaps a more fundamental sort of understanding, that undergirds other kinds of understanding, including explanatory and objectual understanding (Stuart, 2018). Increases in practical understanding are, or are very closely tied to, increases in cognitive abilities, e.g., abilities to create and manipulate models, theories, diagrams, methods, or real systems (Baumberger, 2019; Stuart, 2016, 2018; Le Bihan, 2016; Wilkenfeld, 2013; Regt, 2017; Elgin, 2017; Hills, 2016).

How can the use of imagination required by modernist stories increase cognitive abilities? When scientists tell the story of a breakthrough, they often mention some paper they were reading at the time of the breakthrough, whose significance suddenly became clear to them. E.g., they realized that a certain method or a new piece of information was exactly what they needed to solve a problem of their own (Stuart, 2022). Narrative plays a key role here, because reading papers at a surface level, without imagining what is happening behind the scenes or how a given methodology might be working, or other ways that methodology might be used, would not yield any new abilities to solve their problem, because the papers they are reading *are not about their problem*. The modernist style requires imagination to unpack and comprehend the story. Using our imaginations to fill in the details of the narrative by ourselves makes the story *ours*, it suggests ideas about methods or systems or materials that enable us to solve *our* problems. The result of this process is a new ability, or set of abilities, in the sense that before, we were not able to solve our problem, but now, we can. Before we could not say how this kind of experiment might work on that kind of system, but now, we can. Before we had no facility with concept x or model y, and now, we do. This counts as new practical understanding, and it is specially facilitated by the modernist style, which requires active use of the imagination, through which existing abilities are brought to bear on new problems, or new abilities are created.

## Going further: towards an epistemology of epistemic genres in science

While of the genre of experimental reports might facilitate the attainment of objectual and practical understanding, it is also true that the genre conventions of scientific articles might make possible new kinds of epistemic failures that are not linked to the validity of individual statements or arguments.

At the extreme, someone might claim that being written in a modernist style is precisely the *problem* with today’s scientific writing. Mirroring criticisms of modernist art, one might claim that leaving things up to the reader is simply lazy. The assumption here would be that the author merely *hopes* that the reader will find something of epistemic value in their hastily compiled data. Presenting that data using modernist techniques tells the reader to do the imaginative work themselves, and this saves the author the difficult task of finding and presenting something of value.

We think this is a possibility that deserves to be explored further. There are serious difficulties that people encounter when reading scientific articles, and some of them might be traceable to the misuse of otherwise helpful modernist techniques. We think such a complaint must be made carefully, however. We think that most scientists, like most artists, are not trying to take advantage of their audience. However, we also think that, for example, early-career scientists could rely on modernist techniques more intentionally and effectively if those techniques were made explicit in science education, including a discussion of what separates their good and bad uses. The lack of direct discussion might be part of the reason such techniques occasionally are misused.

Finally, we need not choose either to celebrate or mourn the inclusion of modernist techniques in scientific writing. It would be better to account for *both* the epistemic affordances *and* the epistemic dangers of using modernist conventions. The point we are making is simply that scientific writing *is* fruitfully characterized as modernist, because focusing on the epistemic affordances of that style of writing partially explains why it is that scientists write as they do, and also how scientific articles can be written (or read) poorly.

## Conclusion

In this paper, we argued that the epistemology of scientific articles is incomplete without a narrative perspective that captures the diversity of reporting practices across scientific domains and which makes sense of their epistemic genre. To that end, we considered the case of experimental reports from the hard sciences and argued that their epistemological features are best described by portraying them as employing the same techniques that modernist narratives employ, to address similar aesthetic-epistemic challenges: i.e., presenting data from multiple conflicting perspectives in a way that respects the open-endedness and data-driven nature of science, and requires the use of imagination on behalf of the reader to “complete” the story. Further, we demonstrated how this analogy helps to link the epistemology of experimental reports with objectual and practical understanding via imagination. Finally, we showed that this narrative perspective helps us to think in new ways about the epistemic normativity of scientific articles. With this, we call on philosophers of science to engage further with the epistemology of the genres of scientific articles, and scientific writing more generally.

## Footnotes

1 C.f. Richard Feynman: ‘Science is only useful if it tells you about some experiment that has not been done; it is no good if it only tells you what just went on. It is necessary to extend the ideas beyond where they have been tested’. Quoted in Mitchell (2000).

2 This corresponds with the earlier remarks about scientific articles not merely being arguments. The fact that Suppe’s (1998a) and Hardcastle’s (1999) analysis failed to pinpoint an invariant argumentative structure among scientific reports (along with Suppe’s observation that the majority of statements in scientific papers are observational) speaks in favour of the idea that many experimental papers are engaged in something other than arguing for a single thesis: they are engaged in *reporting* experimental observations.

3 Much of this work builds on a heritage in historiography, philosophy of history, and the philosophy of historical sciences, which analyzes the use of narrative in the practice of identifying and generating historical explanations. See, e.g., Ashworth (2019), Abbott (2008), Barthes and Duisit (1975), Bath (1994), Mink (1970), Roth (1989, 2017), and Ankersmit (1983).

4 The opinion piece generated a worry: such an approach to scientific data could lead to embellishment, concealment, and distortion for the sake of cheap effect, which stands against the spirit of science (Katz, 2013). A settlement was proposed by Bergmann (2013): while storytelling necessitates the exclusion of some data, perhaps that is acceptable, as long as data are not actively hid or oversimplified.

5 Our definition is syncretic and draws on the narrative features discussed by Carroll (2001), Currie (2010), Velleman (2003), Gerrig (1993, 3), Matravers (2014), Davies (2007), and Morgan (2017).

6 Summarised in Kozlov and Nagoshi (2019).

7 Suppe’s case contained twenty-two figures and sub-figures and five tables; two Hardcastle’s (1999) cases have twenty-two and forty-three pieces of original data.

8 We recognize that there will be many relevant differences between the kinds of narratives we find among scientific documents, from case studies to grant applications, research notes, proofs, and so on, and also within the same kind of document in different scientific fields. We focus on the class of documents that report experimental findings produced via research that relies on multiple experimental tools and methods, which includes a great deal, but not everything.

9 The kind of imagination involved in this case seems to be unconscious, automatic imagination. In some cases, especially those related to scientific articles, the kind of imagination required will be conscious, or a combination of both kinds. For the epistemology of these kinds of imagination, see Stuart (2019).

10 Chemist Reuben Hudson teases the idea that the IMRD sections are stages of a dramatic prose, with the Results section being the climax of the “story” (Hudson, 2015).
